# Sero-prevalence of *Toxoplasma gondii* and associated risk factors among psychiatric outpatients attending University of Gondar Hospital, Northwest Ethiopia

**DOI:** 10.1186/s12879-019-4234-6

**Published:** 2019-07-04

**Authors:** Barnabas Achaw, Habtie Tesfa, Ayalew Jejaw Zeleke, Ligabaw Worku, Ayenew Addisu, Niguse Yigzaw, Yalewayker Tegegne

**Affiliations:** 1 Universit of Gondar Hospital, College of Medicine and Health Sciences, University of Gondar, Gondar, Ethiopia; 2 Department of Medical Parasitology, School of Biomedical and Laboratory Sciences, College of Medicine and Health Sciences, University of Gondar, Gondar, Ethiopia; 3 Department of Psychiatric Nursing, Department of Psychiatry, School of Medicine, College of Medicine and Health Sciences University of Gondar, Gondar, Ethiopia

**Keywords:** Toxoplasmosis, Psychiatric, Ethiopia, Prevalence

## Abstract

**Background:**

Toxoplasmosis is caused by an obligatory intracellular coccidian protozoan organism, *Toxoplasma gondii*. It has a worldwide distribution, affecting one-third of the world population. Psychiatric patients have a higher risk of exposure to *Toxoplasma gondii* infection due to lack of good personal hygiene. The burden of toxoplasmosis among psychiatric patients in Ethiopia has not been studied extensively. Thus, the study aimed to determine the seroprevalence of *Toxoplasma gondii* and associated risk factors among psychiatric outpatients at the University of Gondar Comprehensive and Specialized Hospital Psychiatric clinic.

**Methods:**

A cross-sectional study was conducted at the University of Gondar Comprehensive and Specialized Hospital from February to May 2018. Venous blood was collected from 304 study participants (152 psychiatric outpatients and 152 control groups). Anti-*toxoplasma* antibodies were determined using *Onsite* Toxo immunoglobulin G (IgG) and immunoglobulin M (IgM) Rapid Test. A Chi-square test was carried out to compare the two groups and a logistic regression analysis was conducted to check the association between variables. *P-value* less than 0.05 was taken as statistically significant.

**Results:**

The seroprevalence rate of anti-*Toxoplasma gondii* IgG antibodies was 33.6, and 16.4% in the psychiatric outpatients and the control samples, respectively. Anti-*Toxoplasma gondii* IgM sero-prevalence was 1.3% in the former patients and 3.9% in the latter group. Owing cat (AOR = 2.862 95% CI = 1.445–5.666 *P = 0.003*), cleaning cat excreta (AOR = 2.966 95% CI = 1.317–2.652 *P = 0.007*), and farming (AOR = 2.058 95% CI = 1.018–4.163 *P = 0.045*) were found to be significantly associated with the sero-prevalence of anti-*Toxoplasma gondii* IgG antibody.

**Conclusions:**

This study highlighted that, the prevalence of anti-*Toxoplasma gondii* IgG antibodies was significantly higher in psychiatric outpatients than the control group (*p = 0.001*). Cat ownership, cleaning of cat excreta, and farming were found to be statistically significantly associated with the seroprevalence of anti-*Toxoplasma gondii* IgG antibody.

## Background

Toxoplasmosis is a parasitic disease caused by *Toxoplasma gondii.* It is an intracellular protozoan parasite in the phylum Apicomplexan. It has a wide variety of intermediate hosts, including humans and other warmblood mammals [1]. Toxoplasmosis is a major public health problem, as a cause for a high socioeconomic impact including the cost of caring for sick, children with mental disability and blind [2].

In humans, primary infection is usually subclinical, in some patients however cervical lymphadenopathy or ocular disease can be present. Infections acquired during pregnancy may cause severe damage to the fetus. In immune compromised patients, reactivation of latent infection can cause life-threatening encephalitis [3]. The dopamine levels may be affected by *T. gondii*, resulting in alterations in CNS. Previous studies showed that behavior can be affected by latent Toxoplasma infection, possibly being a contributory, or even causative, factor in some psychiatric disorders, including anxiety, depression, and schizophrenia [4, 5].

Besides, Infection with toxoplasma is mostly sub-acute, with signs of focal neurologic which happens along with altered mental state, fever, and headache. Subcortical or cortical lesions of the brain can be present in over half of the patients with toxoplasmosis, resulting in hemiparesis, ambulatory, and difficulty of speaking and walking problems [6].

*T.gondii* has a complex life cycle and is an important foodborne pathogen. The major means of transmission to humans results from the ingestion or handling of undercooked or raw meat containing tissue cysts. On the other hand, the human can get the infection due to direct contact with cats or from the consumption of water or food contaminated by oocysts excreted in the feces of infected cats [7].

IgM and IgG antibodies detection in the patient’s serum is the commonly used method of toxoplasmosis diagnosis. Within a few days to one week of *T. gondii* infection, IgM antibodies can easily be detected. Whereas, IgG antibodies are detected within one up two weeks of its infection, a peak concentration of this antibodies observed after four months, followed by decreasing to lower levels and remaining positive for the rest of the infected person lifespan. Having a positive IgG antibody test result with a negative IgM antibody shows as the patient have chronic *T. gondii* infection. Moreover, an individual having negative IgM antibody test basically excludes acute infection [8].

The prevalence rate of toxoplasmosis varies across different countries, in the world and even among different communities in the same region with in a country. In Ethiopia, the highest prevalence (95.1%) rate of *T. gondii* was reported from Butajira among hospitalized patients found in the age group of 15–49 years [9].

Most of the time, psychiatric patients have a high risk of exposure to *T. gondii* infection due to lack of good personal hygiene, self-care skills, and a tendency to pica. Studies performed in various countries showed increased seroprevalence of toxoplasmosis in psychiatric patients [10]. However, no studies have been conducted in Ethiopia on the burden of toxoplasmosis among psychiatric patients. Therefore, the aim of this study was to determine the prevalence of *T. gondii* infection and associated risk factors among psychiatric patients.

## Methods

### Study area, design and period

An institution based cross-sectional study was conducted among psychiatric patients at the University of Gondar Comprehensive and Specialized Hospital, psychiatric outpatient’s clinic, Gondar, Northwest Ethiopia. Gondar town is located 742 km away from Addis Ababa. Based on the 2007 Ethiopian census report, the population of Gondar town was estimated to be 323,900 [11]. Currently, the town has one referral hospital, University of Gondar Comprehensive and Specialized Hospital, which is a teaching as well as referral hospital. It serves for more than 5 million people. Psychiatric patients with the age of 18 and above and willing to participate in the study were included in the study but pregnant women and Human Immunodeficiency Virus (HIV) positive individuals were excluded. The study was carried out from February 1 to May 30 2018.

### Sample size determination and sampling technique

The sample size was calculated by using Epi.info version 7.1.5.2 by taking a study conducted in Libiya with proportion for the control group p1 = 33% and for case group p2 = 50.3% [12]_._ Therefore, the minimum required sample size was 276. By considering 10% non-response rate total sample size was 304 (152 for each group). A Systematic random sampling technique was used to select the study participants.

### Data collection procedure

#### Questionnaire survey

Data were collected by using pre-tested and semi-structure Amharic version questionnaires. Psychiatric nurses were recruited for data collection and supervised by the principal investigator. The questionnaire had two parts, the first part consisted of socio-demographic characteristics of study participants which includes age, sex, marital status, residence, occupation. The second part of the questionnaire contained of potential risk factors.

### Sample collection and laboratory analysis

Three milliliters of venous blood was collected by trained laboratory technologists and the samples were dispensed into serum sample test tube. One hundred fifty samples from the psychiatric outpatient clinic and one hundred fifty-two samples from VCT (Volunteer Counseling and Testing) clinic were transported to the hospital laboratory and screened for *Toxoplasma gondii* IgG/IgM antibodies. Urine was collected using a clean urine cup from all women study participants to screen pregnancy. Blood samples were centrifuged at 3000 rpm for 5 min using centrifuge (Hettich, Germany) to separate serum portion of the blood component. The laboratory test analysis was done by the principal investigator.

#### Toxoplasma gondii antibody test

Anti**-***Toxoplasma gondii* antibodies- IgG and IgM were determined by using on siteToxo IgG/IgM Rapid Test (CTK BIOTECH, San Diego, USA) which is a lateral flow chromatographic immunoassay for the simultaneous detection of IgG and IgM anti-*Toxoplasma gondii* antibodies in human serum or plasma.

#### Urine HCG test

For excluding pregnant women urine HCG (human chorionic gonadotropin) test was determined for both the cases and control group by using a rapid chromatographic immunoassay test strip (Assure Tech Co Ltd., Hangzhou) which detects the hormone HCG in a urine sample. This hormone is found only in the body during pregnancy.

#### Human immune deficiency virus testing

HIV screening test was carried out using first response, Uni-gold, and vikia HIV 1/2 antibody screening test kit and following the manufacturer’s instruction. The test kit is a visually qualitative immune assay for the detection of antibodies to HIV-1 and HIV-2 in whole blood, plasma or serum.

### Data quality control

The questionnaire was prepared in English, translated to Amharic language. It was pre-tested on eight psychiatric patients and eight volunteers in other similar psychiatric and VCT clinics. One day training was given for data collectors. Manufacturer instructions were strictly followed throughout the laboratory procedures.

### Data processing and analysis

Data were coded, entered, and cleaned using the statistical software Epi info version 7.1.5.2 and exported to the statistical package for social sciences (SPSS) version 20. Frequency tables, figures, and texts were used to present the summarized data. A Chi-square test was used to compare results and logistic regression analysis was conducted to check associations between dependent and independent variables. *P- value < 0.05* was considered as statistically significant.

## Results

### General characteristics of study participants

A total of 304 participants who attended the University of Gondar Comprehensive and Specialized Hospital Psychiatric and VCT clinic from February to May 2018 were included in the study. The subjects were categorized into psychiatric patients and non-psychiatric volunteers. The number of participants in each group was 152. The mean age of the participants was 31 ± 10.2 years, with a minimum age of 18 and a maximum of 70 years. The proportion of males in the case and control groups were 55.9 and 61.2%, respectively. The majority of the participants were Orthodox Christians, 258(84.9%), lived in rural areas, 192(63.2%) and 108 (35.5%) of them were government employees (Table 1).

**Table 1:** Socio demographic characteristics of the study subjects, Northwest Ethiopia, 2018

Variables	Psychiatric group Frequency n(%)	Control group Frequency n (%)	Total Frequency n (%)
Sex			
Male	85 (55.9)	93 (61.2)	178 (58.6)
Female	67 (44.1)	59 (38.8)	126 (41.4)
Age			
≤ 20 21–40	15 (9.9) 98 (64.5)	3 (2.0) 136 (89.5)	18 (5.9) 234 (77.0)
≥ 41	39 (25.7)	13 (8.6)	52 (17.1)
Resident			
Urban	75 (49.3)	37 (24.3)	112 (36.8)
Rural	77 (50.7)	115 (75.7)	192 (63.2)
Occupation			
Student	29 (19.1)	35 (23.0)	64 (21.1)
Government	29 (19.1)	79 (52.0)	108 (35.5)
Daily labor	20 (13.2)	11 (7.2)	31 (10.2)
Farmer	55 (36.2)	18 (11.8)	73 (24.0)
Merchant	9 (5.9)	9 (5.9)	18 (5.9)
Religion			
Orthodox	135 (88.8)	123 (80.9)	258 (84.9)
Protestant	3 (2.2)	11 (7.2)	14 (4.6)
Muslim	12 (7.9)	17 (11.2)	29 (9.5)
Other	2 (1.3)	1 (0.7)	3 (1.0)
Level of education			
Illiterate	55 (36.2)	10 (6.6)	65 (21.4)
Elementary	36 (23.7)	15 (9.9)	51 (16.8)
Secondary	25 (16.4)	19 (12.5)	44 (14.5)
Certificate	36 (23.7)	108 (71.1)	144 (47.4)

### Sero-prevalence of anti *T. gondii* antibodies among cases and control groups

The overall seroprevalence of *T. gondii* infection was 25 and 2.6% for IgG and IgM antibodies, respectively. The seroprevalence of anti-*T. gondii* IgG antibodies in psychiatric patients was 33.6% and it was significantly higher than that of the control group 16.4% (*P = 0.001*). Two (1.3%) of the psychiatric patients and 6 (3.9%) of the control group were IgM positive. There was no statistically significant association in IgM seroprevalence between the psychiatric patients and the control group (Table 2).

**Table 2:** Sero-prevalence of *T. gondii* among psychiatric patients and control group, Northwest Ethiopia, 2018

Variables	Cases (psychiatric patients) n (%)	Control n (%)	X^2^	*P*. value
IgG positive	51 (33.6%)	25 (16.4%)	11.860	0.001*
IgG negative	101 (66.4%)	127 (83.6%)		
IgM positive	2 (1.3%)	6 (3.9%)	2.054	0.143
IgM negative	150 (98.7%)	146 (96.1%)		

*statistically significant at *P*. Value < 0.005

### Risk factors associated with sero-prevalence of *Toxoplasma gondii*

The multivariate logistic regression analysis showed that ownership of cats (AOR = 2.862, CI = 1.445–5.666, *P = 0.003*), cleaning up of cat excretion (AOR = 2.966, CI = 1.317–2.652, *P = 0.007*), and farming (AOR = 2.058, CI = 1.018–4.163 *P = 0.045*) were significantly associated with the seroprevalence of anti-*T. gondii* IgG antibodies.

However, other characteristics such as sex, age group, residence, occupation, religion, a habit of eating uncooked meat and raw vegetables, hand washing habits, source of water, history of blood transfusion and habits of chat chewing did not show any significant association with anti-*T. gondii* IgG antibodies (Table 3).

**Table 3:** Bivariate and multivariate logistic regression analysis on sero-prevalence of *T. gondii* among study participants Northwest Ethiopia, 2018

Variables	Presence of IgG		COR(95% CI)	AOR(95% CI)	*P* value
	Yes	No			
Sex					
Male	42 (23.6%)	136 (76.4%)	0.836(.495–1.411)		
Female	34 (27.0%)	92 (73.0%)	1		
Age					
≤ 20	7 (38.9%)	11 (61.1%)	2.071(.766–5.600)		
21–40	55 (23.5%)	179 (76.5%)	1.727(.559–5.339)		
≥ 41	14 (26.9%)	38 (73.1%)	1		
Resident					
Urban	33 (29.5%)	79 (70.5%)	1.447(.853–2.457)	0.697(.334–1.455)	0.337
Rural	43 (22.4%)	149 (77.6%)	1	1	
Occupation					
Student	20 (31.2%)	44 (68.8%)	2.000(.976–4.099)		
Government	20 (18.5%)	88 (81.5%)	0.955(.380–2.395)		
Daily labor	10 (32.2%)	21 (67.7%)	1.054(.509–2.181)		
Farmer	22 (30.1%)	51 (69.9%)	7.727(.961–62.156)		
Merchant	1 (5.6%)	17 (94.4%)	1.061(.248–4.531)		
Other	3 (30%)	7 (70%)	1		
Level of education	21 (32.3%)	44 (67.7%)	1.145(.517–2.538)		
Illiterate	15 (29.4%)	36 (70.6%)	1.273(.548–2.957)		
Elementary	12 (27.3%)	32 (72.7%)	1.977 (1.018–3.840)		
Secondary	28 (19.4%)	116 (80.6%)	1		
Certificate					
Cat owner					
Yes	56 (39.4%)	86 (60.6%)	4.623 (2.598–8.229)	2.862 (1.445–5.666)	0.003*
No	20 (12.3%)	142 (87.7%)	1	1	
Clean up cat excretion					
Yes	33 (55.9%)	26 (44.1%)	5.962 (3.239–10.977)	2.966 (1.317–2.652)	0.007*
No	43 (17.6%)	202 (82.4%)	1		
Raw meat consumption					
Yes	36 (30.5%)	82 (69.5%)	1.602(.948–2.710)	1.445(.787–2.652)	0.235
No	40 (21.5%)	146 (78.5%)	1	1	
Raw vegetable consumption					
Yes	39 (23.5%)	127 (76.5%)	0.838(.498–1.410)		
No	37 (26.8%)	101 (73.2%)	1		
Farming					
Yes	43 (34.7%)	81 (65.3%)	2.365 (1.394–4.012)	2.058 (1.018–4.163)	0.045*
No	33 (18.3%)	147 (81.7%)	1	1	
History of blood transfusion					
Yes	9 (31.0%)	20 (69.0%)	1.397(.607–3.215)		
No	67 (24.4%)	208 (75.6%)	1		
Habit of chat chewing					
Yes	14 (29.8%)	33 (70.2%)	1.334 (1.671–2.653)		
No	62 (24.1%)	195 (75.9%)	1		

*COR* Crude odds ratio, *AOR* adjusted odds ratio, *CI* Confidence interval, *statistically significant at *P* < 0.05 **statistically significant at *P* < 0.01

### Sero-prevalence of toxoplasmosis among psychiatric patients with different clinical characteristics

Out of the 152 psychiatric patients, almost half (49.3%) were schizophrenic followed by severe depressive episodes (29.6%), and bipolar disorder (13.8%), whereas anxiety was the least (7.2%).

**Fig. 1:**
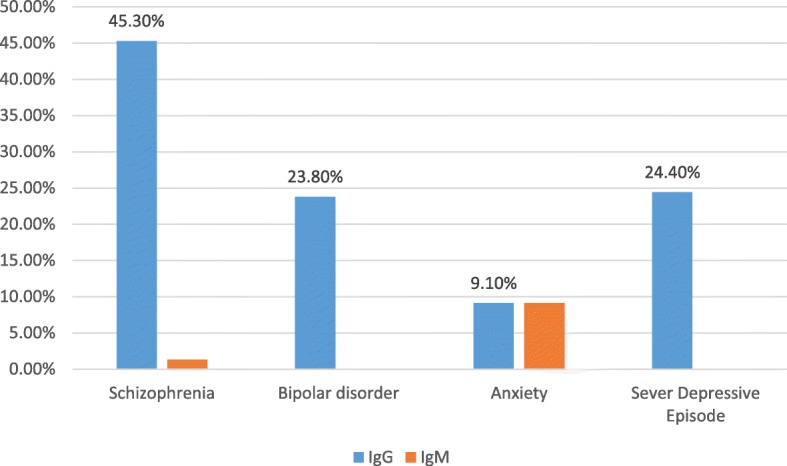
Sero-prevalence for *T. gondii* antibodies in psychiatric patients at University of Gondar psychiatric clinic, Northwest Ethiopia, February to May 2018

In the cases group, the overall seroprevalence of anti *T.gondii* for both IgG and IgM antibodies was highest in schizophrenic patients. The overall sero-prevalence of anti-*T.gondii* among the different types of psychiatric patient’s is shown on Fig. 1.

## Discussion

Although several studies have been conducted on the seroprevalence of *T. gondii* infection in Ethiopia, most of the previous studies focused on pregnant women and immunocompromised patients. Based on our review of literature no evidence of other investigations found related to the current study in Ethiopia; so that the present study was conducted in order to compare the prevalence of *T. gondii* infection between patients with psychiatric disorder and controls, as well as to evaluate the potential association between *T. gondii* infection and associated risk factors in University of Gondar Comprehensive and Specialized Hospital Psychiatric clinic outpatients.

A number of investigations showed that chronic *Toxoplasma* infection may alter human behavior and causes psychiatric disorders. These behavioral changes related to infection with *T. gondii* may be due to the parasite itself, which causes infection with nerve damage. The cytokine IFN- *γ*, is mainly responsible for immunological defense against *T. gondii* is essential in all infected tissues, including the central nervous system [13–15].

Moreover, as *T. gondii* s is a neurotropic organism with a complex mechanism of access to the brain where it encysts in glial cells, neurons, and astrocytes. Damage is characterized by many foci of enlarging necrosis and microglial nodules. It might also incorporate its genome into the cellular Deoxyribonucleic acid with the potential of altering brain function or modifying the growing neuronal cell in vitro [16].

The overall seroprevalence of *T. gondii* infection in this study was 27.6%. The finding is in line with those of previous similar studies carried out in Nigeria (32.1%) [17] and Ahvat, Iran (28.3%) [18]. However, it is lower than those studies done in Libya (41.7%) [12], Western Romania (57.7%) [19] {and Mashhad, Northwest Iran (43.2%) [20]}. On the other hand, the seroprevalence of *T. gondii* antibodies in the present study was higher than studies conducted in Durango city, Northern Mexico (12.5%) [21], India (20.3%) [22] and Mexico (6.1%) [3]. The most possible explanation for such differences in seroprevalence of *T. gondii* infection might be due to differences in culture, socioeconomic status, populations sampled, climatic condition, literacy status of the study participants, personal hygiene, types of the laboratory method used and environmental exposure.

This study showed that the seroprevalence rate for anti-*T. gondii* IgG antibodies in psychiatric patients 51(33.6%) were statistically higher than that in the control group. According to this result, chronic *T. gondii* infection in psychiatric patients 51(33.6%) was higher than in the control group which was found to be 25 (16.4%). This finding was supported by a seroprevalence study conducted in Nigeria (30.7%) IgG antibody in mental disabled patients than the 17.8% in the control group [17]. A study in Tripoli, Libya also found a high (50.3%) seroprevalence rate of IgG antibodies in psychiatric patients than for the 33% in the control volunteers (33%) [12]. The difference in seroprevalence of anti-*T. gondii* IgG between psychiatric patients and controls could be due to the difference in sanitation practices. In addition, most psychiatric patients were from the lower socio-economic level. On the other hand, the seroprevalence of anti-*T. gondii* IgM antibodies in psychiatric patients 1.3% was lower than 3.9% in the control group, even though the difference was not statistically significant (*p-value* = 0.143). Similar findings were reported from Nigeria [17] and Iran [5]. The possible reason for the high seroprevalence of anti-*T. gondii* IgM antibody in the control group compared with the cases may be due to the living of control group in areas that frequently expose people to infections.

In the current study, individuals who had cats (AOR = 2.862, CI = 1.445–5.666, *p = 0.003*) showed a statistically significant association in the seroprevalence of anti-*T. gondii* IgG antibodies. This finding was supported by those of studies conducted in Libya [12], Central Ethiopia [23], and Nazareth town [24]. This is mainly due to the fact that cats are a definitive host for *T. gondii*, and play an important role in the transmission and continuing this parasite in nature [25].

In addition, individuals who frequently cleaned cat excretions showed a statistically significant association with the seroprevalence of anti-*T. gondii* IgG antibodies. The finding was similar to that of a study conducted in Malaysia [26]. One of the causes for this high prevalence could be attributed to the close contact of humans with cats as they excrete millions of oocysts within a short period of time and play a major role in the transmission of *T. gondii* [24]. In our culture cats and dogs have a close proximity to people. Cats are definitive hosts where the sexual multiplication of *T. gondii* takes place [27].

Farming (gardening and agriculture) (AOR = 2.058, CI = 1.018–4.163, *P = 0.045*) was significantly associated with the seroprevalence of anti-*T. gondii* IgG antibody. This is in agreement with a study in Gondar [28] and Bench Maji zone [29]. This might have showed that contact with soil could be potential risk factor for an increasing seroprevalence of anti-*T. gondii* IgG antibodies. This may be due to the fact of that the oocyst of *T. gondii* can remain infective for 12 to 24 months under favorable conditions [30]. In the present study, age and sex didn’t show any statistically significant association with the seroprevalence of *T. gondii* IgG antibody. On the other hand, a study conducted in Mashhad city (Iran), age and sex showed a statistically significant association with the seroprevalence of anti-*T. gondii* IgG antibody in which females and study participants above the age of 40 years were at risk of infection [31]. This variation might be due to differences in type of study population, and study period.

In the present study a high seroprevalence of anti-*T. gondii* IgG antibody was observed among schizophrenic patients (45.3%). This is in-line with a finding reported from Turkey (43.5%) [32]. However, it is higher than the finding of studies conducted in Iran (18.5%) [20], Malaysia (30.7%) [26], and Mexico (26.3%) [21]. It was lower than the result noted in Turkey (66%) [33], Libya (53.4%) [12], and Yemen (53%) [34]. Being high seroprevalence of anti-*T. gondii* antibody among schizophrenic patients may be due to the hypothesis that *T. gondii* is potentially a relevant infection in some cases of schizophrenia [35, 36] or may be related to the fact that patients with schizophrenia have inadequate hygiene and self-care skills, and have a greater tendency to eat contaminated or dirty particles [37].

## Conclusions

In the present study, the seroprevalence of *T. gondii* infection among the psychiatric outpatients was significantly higher than the control group. Cat and dog ownership, cleaning up of cat excretion, and farming (agriculture and gardening) might be the most important routes of *transmission* in our study subjects. The study also found that the prevalence of *T. gondii* infection was higher in schizophrenic patients compared to the other types of psychiatric patients. Thus, we recommend that clinicians should consider screening of psychiatric patients for *T. gindii*.

## Data Availability

I confirmed that all the data for this manuscript are available, if someone wants to request the data can contact Yalewayker Tegegne.

## Abbreviations

AIDS: Acquired Immune Deficiency Syndrome
CNS: Central Nervous System
ELISA: Enzyme Linked ImmunoSorbent Assay
HIV: Human Immunodeficiency Virus
IFAT: Immuno Fluorescence Assay Test
IgA: Immunoglobulin A
IgE: Immuno globulin E
IgG: Immuno globulin G
IgM: Immuno globulin M
LAT: Latex Agglutination Test
PCR: Polymerase Chain Reaction
VCT: Voluntary Counselling and Testing
